# Characterization of a Functional Role of the *Bradyrhizobium japonicum* Isocitrate Lyase in Desiccation Tolerance

**DOI:** 10.3390/ijms160716695

**Published:** 2015-07-22

**Authors:** Jeong-Min Jeon, Hae-In Lee, Michael J. Sadowsky, Masayuki Sugawara, Woo-Suk Chang

**Affiliations:** 1 Department of Biology, University of Texas, Arlington, TX 76019, USA; E-Mails: jeongm@uta.edu (J.-M.J.); hilee@uta.edu (H.-I.L.); 2 Department of Soil, Water & Climate, and The BioTechnology Institute, University of Minnesota, St. Paul, MN 55108, USA; E-Mails: sadowsky@umn.edu (M.J.S.); msugawara@ige.tohoku.ac.jp (M.S.); 3 Division of Biotechnology, College of Environmental and Bioresource Sciences, Chonbuk National University, Iksan 570-752, Korea

**Keywords:** symbiotic nitrogen fixation, *Bradyrhizobium japonicum*, isocitrate lyase (ICL), *aceA*, desiccation stress

## Abstract

*Bradyrhizobium japonicum* is a nitrogen-fixing symbiont of soybean. In previous studies, transcriptomic profiling of *B. japonicum* USDA110, grown under various environmental conditions, revealed the highly induced gene *aceA*, encoding isocitrate lyase (ICL). The ICL catalyzes the conversion of isocitrate to succinate and glyoxylate in the glyoxylate bypass of the TCA cycle. Here, we evaluated the functional role of *B. japonicum* ICL under desiccation-induced stress conditions. We purified AceA (molecular mass = 65 kDa) from *B. japonicum* USDA110, using a His-tag and Ni-NTA column approach, and confirmed its ICL enzyme activity. The *aceA* mutant showed higher sensitivity to desiccation stress (27% relative humidity (RH)), compared to the wild type. ICL activity of the wild type strain increased approximately 2.5-fold upon exposure to 27% RH for 24 h. The *aceA* mutant also showed an increased susceptibility to salt stress. Gene expression analysis of *aceA* using qRT-PCR revealed a 148-fold induction by desiccation, while other genes involved in the glyoxylate pathway were not differentially expressed in this condition. Transcriptome analyses revealed that stress-related genes, such as chaperones, were upregulated in the wild-type under desiccating conditions, even though fold induction was not dramatic (*ca*. 1.5–2.5-fold).

## 1. Introduction

*Bradyrhizobium japonicum* is a soil bacterium that has the ability to form nitrogen-fixing root nodules on soybean (*Glycine max* L.). Because of its symbiotic nitrogen-fixing ability *B. japonicum* has been considered one of the most important microorganisms in sustainable agriculture, as it can improve crop productivity with reduced use of nitrogen fertilizers for plant growth. A number of commercial inoculants of *B. japonicum* have been used for improvement of soybean yield [1]. However, the introduction of inoculants into field soils has not been always successful because of their low survivability under desiccating conditions [2]. Moreover, prolonged exposure to desiccation stress (e.g., drought) can adversely inhibit the nitrogen-fixing ability of *B. japonicum* [3].

Our previous functional genomics studies of *B. japonicum* showed that various stress-responsive genes, encoding heat shock proteins, chaperonins, or sigma factors, were induced by desiccation and desiccation-related stresses [4,5,6]. Interestingly, genes involved in oxidative stress response mechanisms were also induced, indicating that desiccation stress triggers other stress response mechanisms in rhizobia. Severe desiccation may also induce membrane shrinkage and alteration of electron transfer in membranes, leading to generation of reactive oxygen species (ROS). Our preliminary studies showed that there are overlapping gene expression profiles between oxidative and desiccation stresses (unpublished). Among them, *aceA* (blr2455) is of particular interest because it was highly induced under both stress conditions and the encoded protein isocitrate lyase (ICL) is a key enzyme in the glyoxylate pathway, a bypass of the tricarboxylic acid (TCA) cycle. In the glyoxylate cycle, ICL catalyzes the conversion of isocitrate (C_6_) to succinate (C_4_) and glyoxylate (C_2_). Malate synthase (MS) subsequently catalyzes formation of malate (C_4_) from glyoxylate (C_2_) and acetyl-CoA (C_2_) [7]. The glyoxylate cycle is necessary for bacteria to utilize two carbon (C_2_) compounds, such as acetate, for fulfilling carbon requirements [8,9,10].

Several lines of evidence suggest that ICL has additional functions. Firstly, ICL was shown to be involved in bacterial responses to environmental stresses. The *aceA* gene was highly induced by high concentrations of salt in *Shewanella* sp. WP3 [11] and under low temperature in *Colwellia maris* [12]. Secondly, ICL is required for pathogenesis, persistence, and virulence in *Mycobacterium tuberculosis* [13,14]. Deletion of *icl*, a gene analogous to *aceA*, led to reduced persistence of *M. tuberculosis* in mice, indicating that ICL is essential for survival of this bacterium in the host [14,15]. Likewise, *icl* from the phytopathogenic fungus *Leptosphaeria* sp. was highly-induced during its infection of *Brassica napus* cotyledons, and inactivation of the gene resulted in low pathogenicity of the fungus due to its inability to utilize carbon sources provided by the plant [16].

Given the facts that the *B. japonicum aceA* is induced by desiccation and oxidative stresses, and that the genes encoding ICL in other microorganisms are involved in stress tolerance and virulence, it is plausible that *aceA* may play a key role in the survival of *B. japonicum* under various environmental stresses, and perhaps protection of the bacterium from plant defense mechanisms, such as an oxidative burst, during infection.

Here, we performed genetic, transcriptomic, physiological, and phenotyping experiments to determine how *aceA* responds to various stress conditions and how mutations of this gene affect survival and nodulation capacity of *B. japonicum*. In addition, through transcriptomic comparisons between the wild type and the *aceA* mutant, we determined whether ICL altered expression of other genes in this bacterium.

## 2. Results

### 2.1. Purified AceA Has ICL Activity

**Figure 1:**
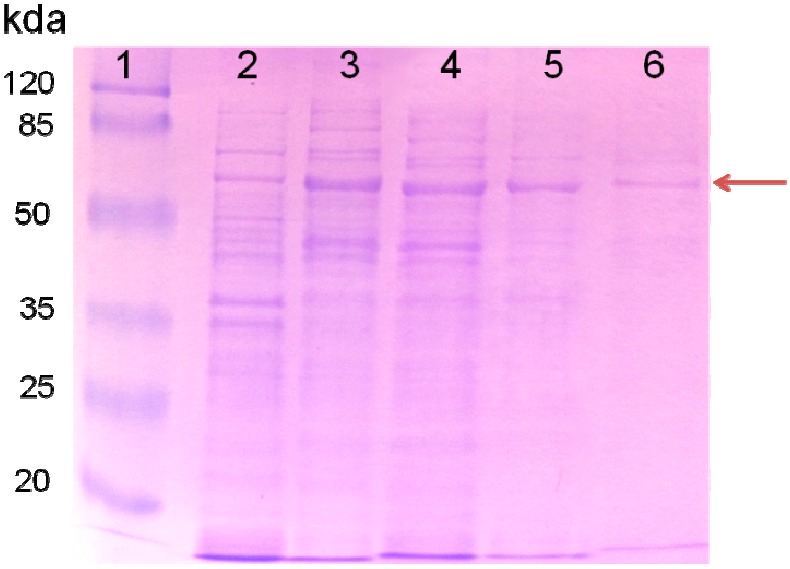
SDS-PAGE of the purified recombinant isocitrate lyase (ICL) protein. The arrow indicates the ICL protein. Lane 1, molecular mass markers (in kDa); lane 2, whole cell extract of *E.coli* RIL (DE3) with control vector pQE2 before induction; lane 3, supernatant of induced *E.coli* RIL (DE3) containing recombinant pQE2 plasmid (pHis-*aceA*) after 0.1 mM IPTG; lane 4, flow through sample after Ni-NTA column; lane 5, washed sample; and lane 6, purified ICL protein. The size of molecular mass markers is indicated on the left.

To confirm that AceA indeed has ICL enzyme activity, AceA was purified, to near homogeneity in a heterologous host, by using the His-tagging method. ICLs have been previously characterized in other microorganisms and ranged from 49–67.5 kDa [13]. The AceA from *B. japonicum* USDA110 is comprised of 592 amino acids and has a predicted molecular mass of approximately 65 kDa. The size of the purified AceA was confirmed by SDS-PAGE (Figure 1). The purified AceA showed minor impurities (Figure 1), which might be due to frame shift during translation as in the case of the ICL homolog from *M. tuberculosis* [17]. The ICL activity of the purified AceA and intermediates during the purification is summarized in Table 1. Although the total activity of the purified AceA decreased by a factor of 360, compared to that of the initial cell-free extract, the specific activity of AceA increased about 2.6 times as the protein amount decreased during purification steps (Table 1).

**Table 1:** Purification of isocitrate lyase enzyme from recombinant *Escherichia coli.*

Purification Steps	Soluble Protein (mg)	Total Activity (U)	Specific Activity (U/mg)
Cell-free extract	45.2	36.2	0.8
Filtered on Ni-NTA column	2.3	3.0	1.3
Purified ICL protein	0.04	0.1	2.1

### 2.2. The aceA Mutant WC2455 Is Sensitive to Desiccation and Salt Stress

**Figure 2:**
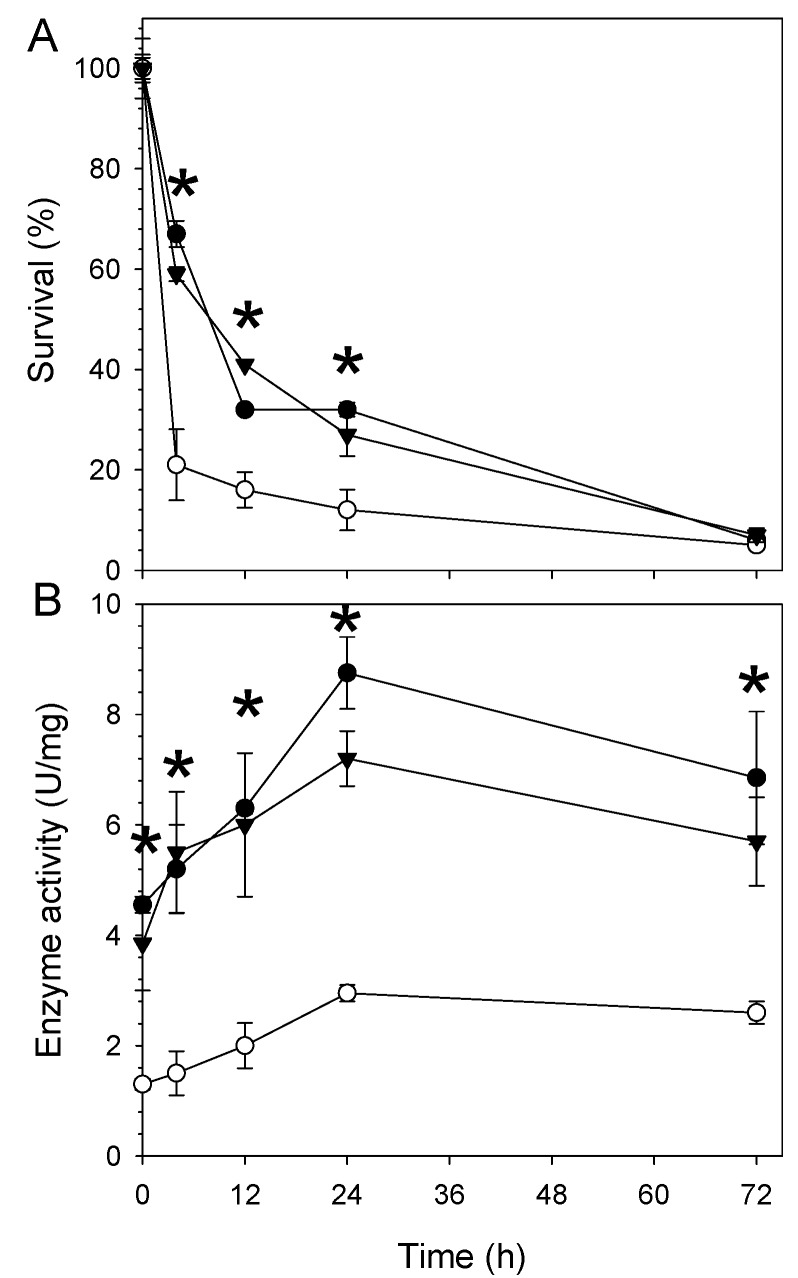
Effect of desiccation-induced stress on (**A**) survival of the three *B. japonicum* strains (wild type, *aceA* mutant WC2455, and *aceA* complemented strain WC2455-C) and (**B**) their ICL enzyme activities. One unit of ICL activity was defined as the amount of enzyme required to produce 1 μmol of glyoxylate per min at pH 6.8 and 30 °C. Symbols: ●, wild type; ○, WC2455; ▼, WC2455-C. Asterisks indicate significant differences between the wild type and mutant strains using *t*-test (*p* < 0.05).

To investigate the role of *aceA* in resistance to desiccation, we compared the survival of wild type USDA110), the *aceA* mutant (WC2455), and the complemented strain (WC2455-C) under desiccation stress conditions. Under fully hydrated conditions (100% relative humidity (RH)), all the strains tested did not show any significant difference in their survival rate (data not shown). In contrast, under desiccation conditions (27% RH), strain WC2455 showed greater sensitivity to the desiccation stress than did USDA110 or WC2455-C (Figure 2A). The survival rate of WC2455 rapidly declined to about 20% after 3 h of desiccation exposure, whereas those of USDA110 and WC2455-C maintained a ~60%–70% survival rate at this time point (Figure 2A). Viability of all tested strains decreased by more than 90% after 72 h exposure to desiccation stress (Figure 2A).

**Figure 3:**
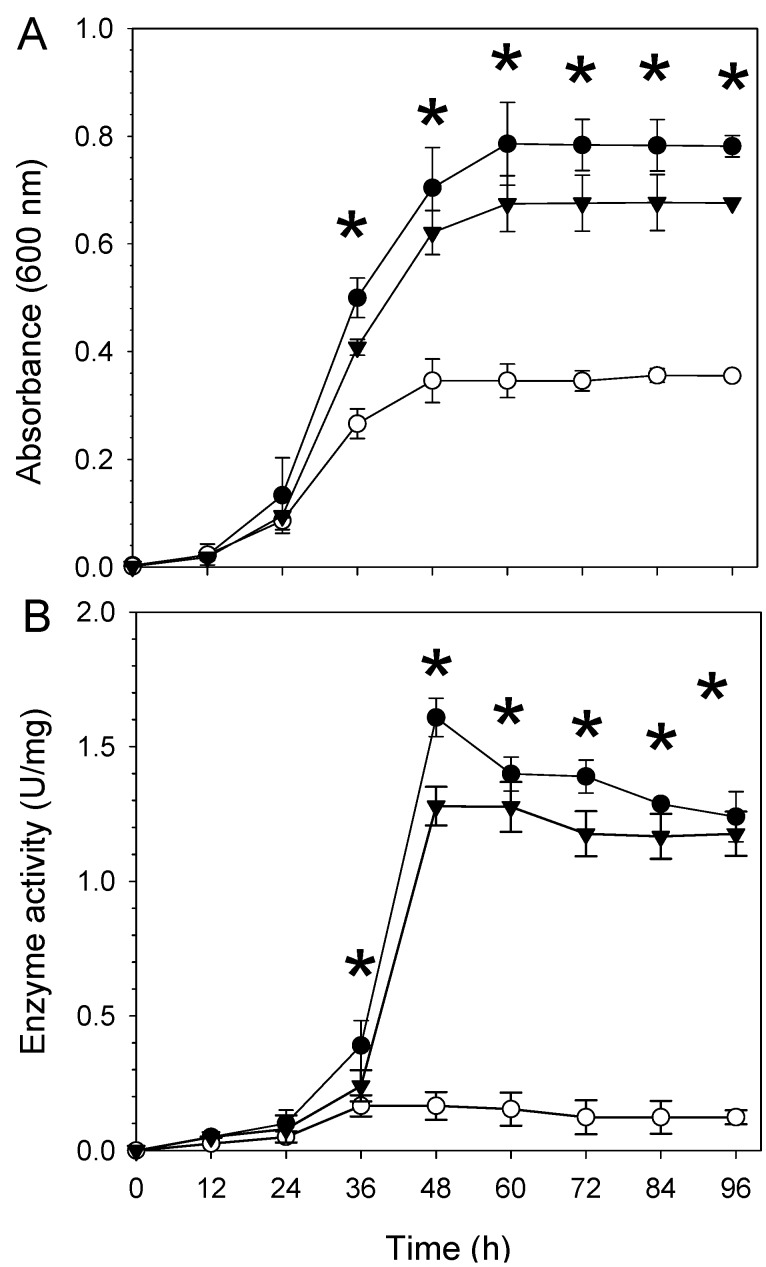
Effect of salt stress (70 mM NaCl) on (**A**) growth of the three *B. japonicum* strains (wild type, *aceA* mutant WC2455, and *aceA* complemented strain WC2455-C) in AG medium and (**B**) their ICL enzyme activities. One unit of ICL activity was defined as the amount of enzyme required to produce 1 μmol of glyoxylate per min at pH 6.8 and 30 °C. Symbols: ●, wild type; ○, WC2455; ▼, WC2455-C. Asterisks indicate significant differences between the wild type and the mutant using *t*-test (*p* < 0.05).

Desiccation stress is also likely to increase salinity in soil environments due to evaporation, and the increased salinity might consequently induce osmotic stress. Thus, we also tested the survival of *B. japonicum* under salt stress conditions. When grown with 70 mM NaCl, wild type USDA110 showed a saturated cell density of ~0.8 OD_600 nm_, whereas the cell density of the *aceA* mutant did not exceed half of that of the wild type strain (Figure 3A). The ICL activity of the wild type and complemented strains (WC2455-C) was about 1.0 and 1.5 U/mg protein during late log phase and stationary phase, respectively, while that of WC2455 was <2 U/mg protein (Figure 3B).

### 2.3. Involvement of AceA in Response to Desiccation Stress Is Independent of the Glyoxylate Pathway or the TCA Cycle

Since ICL is a key enzyme in the glyoxylate pathway, we hypothesized that the glyoxylate pathway or the related TCA cycle pathway may play a role in desiccation stress. Differential expression of genes involved in the glyoxylate pathway and TCA cycle was analyzed in wild type USDA110 and the *aceA* mutant grown under desiccation stress by using qRT-PCR. Only *aceA* was highly expressed by desiccation stress in the wild type, whereas the expression of other genes was modest at best (Table 2). This result indicates that ICL is involved in the response to desiccation regardless of the glyoxylate or TCA cycles.

**Table 2:** Comparison of gene expression level changes against desiccation stress between wild type and WC2455 by qRT-PCR analysis.

Locus (Gene)	Gene Description ^a^	Desiccation	
		Wild Type	WC2455
bll0452 (*suc*A)	alpha-ketoglutarate dehydrogenase	1.0	1.0
bll0455 (*suc*C)	succinyl-CoA synthetase beta chain	−1.3	−1.7
bll1474 (*glcB*)	malate synthase	−1.6	1.1
blr0512 (*sdh*C)	succinate dehydrogenase cytochrome	1.3	1.0
blr2455 (*ace*A)	isocitrate lyase	148.0	ND ^b^
blr5747 (*icd*A)	isocitrate dehydrogenase	−1.3	1.1
blr6519 (*fum*C)	fumarase C	1.6	1.2

^a^ Gene descriptions represent third level annotations from the three-tiered functional level system of *B. japonicum* (www.kazusa.or.jp/rhizobase/Bradyrhizobium/cgi-bin/category_brady.cgi); ^b^ Not detected.

### 2.4. Comparison of the Global Transcription Profiles in Wild Type and Mutant Strains

Since strain WC2455 was significantly more sensitive to desiccation stress than wild type USDA110, genes related to expression of *aceA* may be involved in stress responses. To address this role of AceA, we performed genome-wide transcriptional analyses of wild type USDA110 and the *aceA* mutant (WC2455) after 24 h of desiccation treatment (27% RH).

Microarray analyses showed that 81 genes were differentially expressed, at a 1.5-fold cut-off (Supplemental Table S1). Of these 81 genes, 73 were up-regulated and eight were down-regulated. The upregulated genes were involved in the functional categories of cellular processes, energy metabolism, translation and transport, and binding proteins. Genes related to heat shock response, blr4637 and bll5219 (*hspD*), and seven chaperones encoded by blr4635 (*groEL*), blr4653 (*dnaJ*), blr5625 (*groES*), and blr5626 (*groEL*) were among the highly induced genes in the cellular processes category These genes are related to stress response defense mechanisms [18]. Also, several translation factors that are involved in ribosomal proteins were slightly induced (Table 3). To protect damaged cells from stress, translational machinery presumably associated with AceA could be enhanced. Similarly, more translation-related genes were highly induced in rich medium compared with minimal medium [19,20].

**Table 3:** Changes in expression of physiological process genes in desiccation-grown *B. japonicum*.

Physiological Process	Locus (Gene ID) ^a^	Description ^b^	Fold Induction
Chaperones	bsl3986 (*cspA*)	cold shock protein	1.7
	blr4637	probable HspC2 heat shock protein	1.6
	blr4635 (*groEL*)	chaperonin GroEL	1.5
	blr4653 (*dnaJ*)	molecular chaperone DnaJ family	1.7
	bll5219 (*hspD*)	small heat shock protein	2.1
	blr5625 (*groES*)	10 KD chaperonin	2.5
	blr5626 (*groEL*)	60 KDA chaperonin	2.1
Energy metabolism	blr1656	putative glycosyl hydrolase	2.0
	bll3998	probable succinate-semialdehyde dehydrogenase	2.3
	blr4657	beta-glucosidase	1.6
	bll4784	aldehyde dehydrogenase	1.8
	blr6128 (*cycB*)	cytochrome c552	1.5
	blr7040 (*napC*)	cytochrome C-type protein	2.1
Heat shock response systems	blr0678	heat shock protein 70	2.0
Translation	bll5377 (*rpsK*)	30S ribosomal protein S11	1.8
	bll5381 (*rplO*)	50S ribosomal protein L15	1.6
	bsl5382 (*rpmD*)	50S ribosomal protein L30	1.6
	bsl5391 (*rpsQ*)	30S ribosomal protein S17	1.5
	bsl5392 (*rpmC*)	50S ribosomal protein L29	1.8
	bll5397 (*rplB*)	50S ribosomal protein L2	1.7
	bll5415 (*rplK*)	50S ribosomal Protein L11	2.4

^a^ The differentially-expressed genes from each functional category were chosen with 1.5-fold cut-off and *p* ≤ 0.05; ^b^ Description (annotations) represents the third level from the three-tiered functional level system of *B. japonicum* (www.kazusa.or.jp/rhizobase/Bradyrhizobium/cgi-bin/category_brady.cgi).

### 2.5. The Inactivation of AceA Results in Delayed Soybean Nodulation

**Figure 4:**
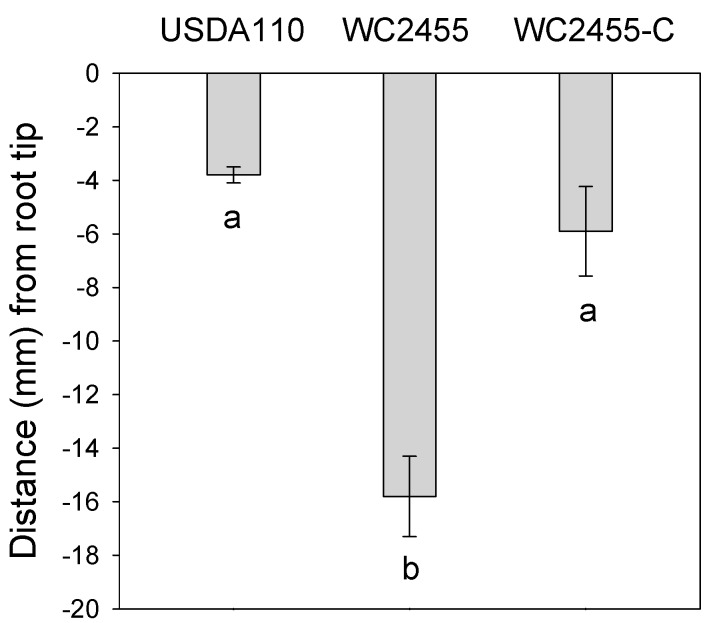
The effect of *ace*A gene on nodulation of *B. japonicum*. The average distance (mm) of nodules form root tip (RT). Values are means ± standard errors of the means from nine replications. The different letters below the bars indicate that the data are significantly different from each other (*p* < 0.05, JMP^®^ 8 software (SAS Institute Inc., Cary, NC, USA).

To evaluate the role of *aceA* in the nodulation efficiency of *B. japonicum*, we first performed nodulation assays under hydroponic conditions using plant growth pouches. No significant difference was found in the number of nodules produced by wild type USDA110, the *aceA* mutant, or the complemented strain. They each formed approximately eight to nine nodules per plant. However, nodules formed by the *aceA* mutant were located most distantly from the initial root tip (RT) marked position compared to wild type USDA110 or the complemented strain, indicating that the mutant had a delayed nodulation response (Figure 4). This result suggests that AceA might facilitate the initiation of nodulation, but may be not essential for soybean nodulation by *B. japonicum*. While the *aceA* mutant showed a delayed nodulation response in growth pouches, no significant difference in the nitrogenase activity, as determined by the acetylene reduction assay, was found between wild type USDA110 and the *aceA* mutant (data not shown).

## 3. Discussion

In this study, we showed that *aceA* is essential for the survival of *B. japonicum* under desiccation stress (Figure 2). There have been several lines of evidence supporting the hypothesis that *aceA* may play an important role in bacterial resistance to various environmental stresses. The *aceA* was induced by high salinity and under acid shock conditions in *Shewanella* sp. WP3 and *M. tuberculosis*, respectively [11,21]. Our previous study showed that H_2_O_2_-mediated oxidative stress also induced the expression of *aceA* [6]. Likewise, it appears that environmental stresses also induce ICL activity in other bacteria. For example, the ICL activity in *Pseudomonas* was increased more than four-fold in an aluminum-rich environment [22]. Enhanced ICL activity was also observed in *Cladosporium sphaerosperum* in the presence of salt [23].

Inactivation of *aceA* did not affect the expression of genes related to either the TCA or glyoxylate cycles under desiccation stress conditions (Table 2). This indicates that *aceA* independently responds to desiccation stress, without interacting with substrates produced by enzymes in these other cycles. Although *B. japonicum* blr2455 was annotated as AceA, according to NCBI BLAST results, the protein specifically contains the ICL/PEPM superfamily conserved domain (cd00377), whose members catalyze either P–C or C–C bond formation/cleavage. Known members that belong to this conserved domain family are phosphoenolpyruvate mutase (PEPM), phosphonopyruvate hydrolase (PPH), carboxyPEP mutase (CPEP mutase), oxaloacetate hydrolase (OAH), isocitrate lyase (ICL), and 2-methylisocitrate lyase (MICL). Thus, the *B. japonicum* AceA appears to be a member of the canonical isocitrate lyase superfamily.

To determine if AceA regulates the expression of other genes, we compared the complete transcription profile of genes in the *aceA* mutant with that of wild type USDA110 during exposure to desiccation stress. Since transcriptome profiles were not much different between the *aceA* mutant and wild type USDA110 under the tested conditions (only 81 genes were differentially expressed with 1.5-fold cut off), our data strongly suggests that AceA likely plays a limited role in overall gene regulation in *B. japonicum*. In addition, we compared these data to previous transcriptome profiles of the wild type USDA110 strain under desiccation-induced stress conditions [4]. Of the 73 up-regulated genes, 28 overlapped, including five translation-related genes (bll5381, bsl5382, bsl5391, bsl5392, and bll5397), five energy metabolism genes (bll3998, bll4784, bll5655, blr6128, and blr7040), four chaperonins (blr4637, blr4653, blr5625, and blr5626), one transport (bsr4636), one nitrogen fixation (blr2764), one regulatory gene (blr2475), and one chemotaxis (bll6865). Interestingly, there were 10 hypothetical function genes detected as overlapped genes. Although some genes were differentially expressed between the two different strains, the fold change was only modest (Table 3). While *aceA* was down-regulated in the mutant strain, there was only a 2.7-fold change (Supplemental Table S1). This might be due to selection of our microarray probe for *aceA*, *w*hich was located almost at the beginning of the translational region for *aceA*, while the inactivated region of *aceA* in the mutant was in the middle of the gene. Thus, a considerable amount of cDNA synthesized from partially transcribed mRNA for *aceA* might have hybridized to the probe.

Our results strongly suggest that *aceA* may play both physiological and functional roles in bacterial stress responses. These observations also raise the possibility that *aceA* in *B. japonicum* may independently play a protective role and has an enzymatic function in the TCA cycle in response to stress responses, such as H_2_O_2_-mediate oxidative stress and desiccation stress.

## 4. Experimental Section

### 4.1. Bacterial Strains and Culture Conditions

The bacterial strains and plasmids used in this study are shown in Table 4. The *B. japonicum* strains were grown aerobically at 30 °C on arabinose-gluconate (AG) medium [24]. The *aceA* mutant (WC2455) [25] and its complemented strain (WC2455-C) [6] were described previously. *Escherichia coli* strains were cultured on LB medium at 37 °C, with shaking at 200 rpm. Antibiotics were used at the following concentrations, when needed: chloramphenicol, 30 µg·mL^−1^; kanamycin, 150 µg·mL^−1^; tetracycline 100 µg·mL^−1^ for *B. japonicum;* and ampicillin, 50 µg·mL^−1^; kanamycin, 50 µg·mL^−1^; and tetracycline, 50 µg·mL^−1^ for *E. coli*.

### 4.2. Desiccation Stress Assay

The survivability of *B. japonicum* strains under desiccation-stress conditions was determined by the filter disk method as previously described [4]. A single colony of each *B. japonicum* strain (USDA110, WC2455, and WC2455-C) was inoculated into 5 mL of AG liquid medium, and cultures were grown to mid-exponential phase and transferred into fresh AG medium (200 mL) to OD_600_ = 0.5. The culture was divided into eight 25 mL aliquots and distributed into 50 mL conical tubes. Cultures were centrifuged at 10,000× *g*, washed, and resuspended in 25 mL minimal medium [26] to give OD_600_ = 0.5. The cell suspension was filtered onto sterilized 0.45 µm cellulose nitrate membrane filters (47 mm diameter, Sterlitech, Kent, WA, USA). The membrane filters were aseptically placed at the bottom of perforated polystyrene petri dishes and immediately transferred into desiccators, which maintained 27% or 100% relative humidity (RH), for desiccation or control conditions, respectively. After incubation in the dark at 30 °C for 4, 24, and 72 h, viable counts of cells on membranes were determined by dilution plating on the agar medium.

**Table 4:** Bacterial strains and plasmids used in this study.

Strain or Plasmid	Genotypes, Relevant Characteristics	Source
*B. japonicum* strains		
USDA110	wild type	USDA-ARS (Beltsville, MD, USA)
WC2455	*aceA*::Km	[25]
WC2455-C	WC2455 complemented strain	[6]
*E. coli* strains		
DH5α	supE44 ∆lacU169 (ø 80lacZ∆M15) hsdR17 recA1 endA1 gyrA96 thi-1 relA1	[27]
RIL(DE3)	argU (AGA, AGG), ileY (AUA), leuW (CUA)	Agilent (La Jolla, CA, USA)
Plasmids		
pTE3	complementing plasmid, Tc^r^	[28]
pRK2073	RK2, Tra^+^,Sm^r^	[29]
pGEM-T easy	cloning vector	Promega (Madison, WI, USA)
pQE2	expression vector, 6X His tag, T7 promoter	Qiagen (Valencia, CA, USA)
pGEM-T easy::*aceA*	pGEM T easy containing 1.5 kb fragment including entire *aceA* gene	This study
pTE-*aceA*	pTE3 containing 1.5 kb fragment of *aceA*	This study
pHis-*aceA*	pQE2 containing 1.8 kb fragment of *aceA*	This study

### 4.3. Salt Stress Assay

The *B. japonicum* cells were grown in AG medium supplemented with 70 mM NaCl. Cell turbidity was measured and monitored at OD_600_ every 12 h, until 96 h. Control cultures were treated with sterilized distilled water (ddH_2_O). Each data point contained three biological replicates.

### 4.4. RNA Isolation and Microarray Analysis

Total RNA was extracted from *B. japonicum* cells by using the hot-phenol method as described previously [30]. Each 100 mL culture of *B. japonicum* strains USDA110 and WC2455 were grown at 30 °C until OD_600_ = 0.5. Cultures were divided into four 25 mL conical tubes, washed, resuspended in minimal medium, and filtered onto cellulose-nitrate membrane filters. Replicate membranes were transferred into desiccators at 27% or 100% RH, and incubated at 30 °C for 0 and 24 h. Cells were recovered from membrane by agitation in 10 mL of medium, centrifuged at 8000× *g*, and washed in minimal medium. All extracted RNA samples were further purified using RNeasy Mini Kits (Qiagen, Germantown, MD, USA) for microarray and quantitative reverse transcription PCR (qRT-PCR) experiments. The synthesis of cDNA synthesis, labeling with Cy3 and Cy5, and microarray hybridizations were performed as described previously [19]. All microarray experiments were performed with three biological replicates for each condition. The raw microarray data from this study are deposited in the NCBI Gene Expression Omnibus (GEO) database under accession number GSE69999 (http://www.ncbi.nlm.nih.gov/geo/query/acc.cgi?acc=GSE69999).

### 4.5. qRT-PCR Analysis

The qRT-PCR was performed according to methods described previously [19]. Gene specific primers used for qRT-PCR are listed in Table 5. For normalization of the qRT-PCR data, the expression value of each gene was calculated relative to *parA* gene (bll0631), encoding the chromosome-partitioning protein. All qRT-PCR experiments were performed with three biological replicates for each condition.

**Table 5:** Primers used for qRT-PCR analysis.

Gene	Forward Sequence 5′–3′	Reverse Sequence 5′−3′
bll0452	CGGCATCGACGACATCTACCTGAT	TCCAGATAGGGCTCGATGAAGTGC
bll0455	GAGACAGAGGAAGACGCCAAGGAA	GCCATGCCGTAGAGCTTGATGATG
bll1474	GCCTCCAAGCGCATCATGTTCATC	CATGTCGACGTTCCAGTCCTCGTA
blr0512	TTCAAGGCCAATGAGCGCGAAG	ACGATCCAGATCAGCACCGTCA
blr2455	GGCGACCAGTACAACAGCTT	GTCTCGATCCAGAGCAGGTC
blr5747	TGTCGACCAAGAACACCATCCTCA	TAGTTCTTGCAGGCCCAGACATAGC
blr6519	GGCCATTTCGAGCTCAACGTCTAC	CTGACGCAATGTTCGGTGAAGGAG
bll0631	TCAACCTTCTGACGGTGAACGC	TGCAGCAATTGCGACAGACCTT

### 4.6. ICL Enzyme Assay

*B. japonicum* cells recovered after desiccation or osmotic treatments were harvested by centrifugation for 5 min at 8000× *g*, and cell pellets were washed in 50 mM Tris-sodium EDTA (TNE) buffer (pH 7.0). Cells were resuspended in 1 mL breaking buffer (20 mM TNE buffer (pH 7.0), 100 mM NaCl, 5 mM MgCl_2_, 0.4 mM EDTA, 1.5 mM DTT and 2% (*wt*/*v*) glycerol) [31] and were subsequently sonicated for 1 min. The cell pellet was removed by centrifugation at 8000× *g* for 5 min. The isocitrate lyase assay was performed according to Sigma-Aldrich’s protocol and the method of Chell [32], with some modifications. A 0.1 mL aliquot of freshly prepared cell-free supernatant was added to the reaction mixture and incubated for 5 min at 30 °C. The reaction mixture contained 30 mM imidazole buffer (pH 6.8), 5 mM MgCl_2_, 1 mM EDTA, 4 mM phenylhydrazine hydrochloride (Sigma-Aldrich, St. Louis, MO, USA), and 1 mM dl-isocitric acid (Sigma-Aldrich, St. Louis, MO, USA). The enzyme activity was determined by measuring absorbance at 340 nm. One unit (U) of the ICL activity was defined as the amount of substrate consumed to produce 1 µmol of glyoxylate per min at 30 °C.

### 4.7. Construction of pQE2::AceA Strain

A 1.8 kb DNA fragment, including the *aceA* coding region, was amplified by PCR using primers *aceA_*fw (5′-TATACATATG**CAA**TTACATG ACATCACCAATAAA-3′) and *aceA*_rv (5′-ATATAAGCTTTTAGCCGAACTGGTTCATCGT-3′), where the underlined sequences indicate *NdeI* and *HindIII* restriction enzyme linkers for *aceA*_fw and *aceA*_rv, respectively. The three nucleotides in bold in *aceA*_fw represent the codon for glutamine placed to avoid a frame shift of the *aceA* coding region. The PCR product was sequenced to confirm the *aceA* coding region and cloned into pQE2 (Qiagen Science, Maryland, MD, USA) to express His_6_-tagged AceA in *E. coli* RIL(DE3) (Agilent, La Jolla, CA, USA). The resulting plasmid was named pHis-*aceA* (Table 4).

### 4.8. Purification of AceA Protein

The AceA was purified by the His-tag method. A single colony of *E. coli* RIL(DE3) containing pHis-aceA was grown overnight in 5 mL LB medium supplemented with ampicillin (50 µg·mL^−1^) and gentamycin (20 µg·mL^−1^). The overnight culture was diluted (1% *v*/*v*) into 50 mL LB medium without antibiotics, and grown for 3 h at 30 °C with shaking at 200 rpm. The subculture was immediately transferred into a pre-chilled 50-mL Erlenmeyer flask, and expression of aceA was induced by adding IPTG to a final concentration of 0.1 mM. After induction for 36 h at 10 °C with shaking, cells were harvested by centrifugation at 4000× *g* for 20 min. The cell pellet was resuspended in 3 mL lysis buffer (50 mM NaH_2_PO_4_, 300 mM NaCl and 10 mM imidazole; pH 8.0) and stored at −80 °C until used.

After thawing the cell suspension at 4 °C, cells were lysed by six repetitions of sonication on ice for 10 s with 10-s pause at each interval. The lysate was centrifuged for 3 min at 4000× *g* at 4 °C and the supernatant was loaded onto a 5-mL Ni-NTA column equilibrated with lysis buffer. The resin was washed twice with wash buffer (lysis buffer containing 20 mM imidazole instead of 10 mM imidazole) and the protein was eluted in 5 mL of elution buffer (wash buffer containing 250 mM imidazole instead of 20 mM imidazole). The eluate was stored as a 10% glycerol solution at −80 °C until used for further experiments. The ICL activity of AceA was measured by the method described above, but using the purified protein instead of the cell-free extract.

### 4.9. Nodulation Assay

Soybean nodulation assays were done in plastic growth pouches (Mega International, St. Paul, MN, USA) as described previously [33]. Soybean seeds were sterilized by washing in 20% solution of commercially available bleach for 10 min, rinsed 3 times with sterile distilled water, washed in 0.01 N HCl for 10 min, and rinsed 3 times with sterile distilled water. Sterilized soybean seeds were placed onto the surface of pre-soaked Whatman filter paper in petri dishes and germinated for 3 days in the dark at room temperature. Three seedlings were aseptically transferred into each autoclaved growth pouch and the position of root tip (RT) was marked on the surface of the growth pouch [34]. Each plant was inoculated with 1 mL of cell suspension of the *B. japonicum* culture prepared by harvesting at mid-log phase, washing with half strength B&D medium (no nitrogen source) [35], and resuspending in the same medium to give OD_600_ = 0.1 (*ca*. 10^8^ cells/mL). The plants were grown in a plant growth chamber at 26 °C with 15 h-day and 9 h-night cycles. Soybean plants were watered with 10 mL of half strength B&D medium for 4 weeks until they were harvested for the following analysis: The number of nodules on the primary root was counted and the distance of the nodules was measured from the RT mark.

## 5. Conclusions

Our findings show the physiological and functional role of *B. japonicum* ICL under desiccation-induced stress conditions. These observations raise the dual function possibility of *aceA* gene in *B. japonicum*: (i) AceA protein independently plays a protective role against environmental factors such as desiccation and salt stresses and (ii) has an enzymatic function in the glyoxylate pathway, a bypass of the TCA cycle, to catalyze the conversion of isocitrate to succinate and glyoxylate.

## Supplementary Materials

Supplementary materials can be found at http://www.mdpi.com/1422-0067/16/07/16695/s1.
