# Supplementary material for: Characterization of a Functional Role of the Bradyrhizobium japonicum Isocitrate Lyase in Desiccation Tolerance
Source: Int J Mol Sci. 2015 Jul 22;16(7):16695–709. doi: 10.3390/ijms160716695 (PMC4519974; doi:10.3390/ijms160716695)
Supplement: Supplementary file 1 [file ijms-16-16695-s001.pdf]

Supplementary Information

Table S1. Functional classification of genes differentially expressed in desiccation stress (RH 27 %, USDA110 *vs.* WC2455). The cut-off threshold was 1.5-fold with q value less than 5%. Positive values in fold change indicate up-regulation while negative values indicate down-regulation. Functional levels were adopted from the rhizobase (http://genome.kazusa.or.jp/rhizobase/Bradyrhizobium/genes/category).

Locus ID	Fold Change	*q*-Value (%)	Functional Level I	Functional Level II	Functional Level III (Description)
bll7086	+1.5	0.00	Biosynthesis of cofactors, prosthetic groups, and carriers	Cobalamin, heme, phycobilin and porphyrin	anaerbic coproporphyrinogen III oxidase
blr4700	+1.7	0.00	Cell envelope	Membranes, lipoproteins and porins	putative outer-membrane immunogenic protein precursor
blr5625	+2.5	0.00	Cellular processes	Chaperones	10 KD chaperonin
bll5219	+2.1	0.45	Cellular processes	Chaperones	small heat shock protein
blr5626	+2.1	0.00	Cellular processes	Chaperones	60 KDA chaperonin
bll6865	+1.9	0.45	Cellular processes	Chemotaxis	flagellin
blr7740	+1.9	0.00	Cellular processes	Chaperones	small heat shock protein
bll7146	+1.7	0.45	Cellular processes	Cell division	metalloprotease
bsl3986	+1.7	0.45	Cellular processes	Chaperones	cold shock protein
blr4653	+1.7	0.00	Cellular processes	Chaperones	molecular chaperone DnaJ family
blr4637	+1.6	0.00	Cellular processes	Chaperones	probable HspC2 heat shock protein
bll3872	+1.6	0.00	Cellular processes	Protein and peptide secretion	HlyD family secretion protein
blr4635	+1.5	0.00	Cellular processes	Chaperones	chaperonin GroEL
blr2767	+1.6	0.45	Central intermediary metabolism	Nitrogen fixation	iron-sulfur cluster-binding protein
blr2764	+1.6	0.00	Central intermediary metabolism	Nitrogen fixation	cytochrome-c oxidase
bll0823	+1.7	0.00	DNA replication, recombination, and repair	-	DNA gyrase subunit B
bll3998	+2.3	0.00	Energy metabolism	Amino acids and amines	probable succinate-semialdehyde dehydrogenase [NADP+]
blr7040	+2.1	0.00	Energy metabolism	Respiration	cytochrome C-type protein
blr1656	+2.0	0.00	Energy metabolism	Sugars	putative glycosyl hydrolase
bll4784	+1.8	0.45	Energy metabolism	Pyruvate and acetyl-CoA metabolism	aldehyde dehydrogenase

Table S1. *Cont.*

Locus ID	Fold Change	*q*-Value (%)	Functional Level I	Functional Level II	Functional Level III (Description)
blr4657	+1.6	0.45	Energy metabolism	Sugars	beta-glucosidase
blr6128	+1.5	0.00	Energy metabolism	Respiration	cytochrome c552
blr2455	−2.7	0.5	Energy metabolism	TCA cycle	isocitrate lyase
bll1200	+1.8	0.00	Fatty acid, phospholipid and sterol metabolism	-	5-aminolevulinic acid synthase
blr1812	+2.4	0.00	Other categories	Symbiosis	nodulation protein
bll8199	+2.0	0.00	Other categories	Transposon-related functions	putative transposase
bll4642	+1.9	0.00	Other categories	Transposon-related functions	putative transposase
bll7981	+1.7	0.00	Other categories	Other	putative dehydrogenase
bll2019	+1.7	0.00	Other categories	Symbiosis	transcriptional regulatory protein
bll5655	+1.6	0.00	Other categories	Other	alcohol dehydrogenase
bll4232	+1.5	0.00	Other categories	Transposon-related functions	putative transposase
bll0932	−1.6	0.45	Other categories	Drug and analog sensitivity	multidrug resistance protein A
blr3678	−1.6	0.45	Other categories	Other	putative oxidoreductase
blr2475	+1.9	0.45	Regulatory functions	-	tryptophan-rich sensory protein
bll0933	−2.0	0.45	Regulatory functions	-	transcriptional regulatory protein
bll5415	+2.4	0.45	Translation	Ribosomal proteins	50S ribosomal Protein L11
bsl5392	+1.8	0.00	Translation	Ribosomal proteins	50S ribosomal protein L29
bll5377	+1.8	0.00	Translation	Ribosomal proteins	30S ribosomal protein S11
bll5397	+1.7	0.00	Translation	Ribosomal proteins	50S ribosomal protein L2
bll5381	+1.6	0.00	Translation	Ribosomal proteins	50S ribosomal protein L15
bsl5382	+1.6	0.00	Translation	Ribosomal proteins	50S ribosomal protein L30
bsl5391	+1.5	0.00	Translation	Ribosomal proteins	30S ribosomal protein S17
blr7037	+1.9	0.00	Transport and binding proteins	-	periplasmic nitrate reductase
bsr7036	+1.9	0.00	Transport and binding proteins	-	periplasmic nitrate reductase protein
bsr4636	+1.7	0.00	Transport and binding proteins	-	putative cation transport regulator
bll6911	−1.5	0.00	Transport and binding proteins	-	ABC transporter ATP-binding protein

Table S1. *Cont.*

Locus ID	Fold Change	*q*-Value (%)	Functional Level I	Functional Level II	Functional Level III (Description)
bsl2070.3n	+1.6	0.00		-	hypothetical protein bsl2070.3n
blr1806	+2.1	0.00	Hypothetical	No similarity	hypothetical protein blr1806
blr0678	+2.0	0.00	Hypothetical	Conserved hypothetical protein	heat shock protein 70
bll1877	+1.9	0.45	Hypothetical	No similarity	hypothetical protein bll1877
bll1848	+1.9	0.00	Hypothetical	No similarity	hypothetical protein bll1848
bll6880	+1.9	0.00	Hypothetical	No similarity	hypothetical protein bll6880
blr4641	+1.9	0.00	Hypothetical	No similarity	hypothetical protein blr4641
bsr6521	+1.8	0.00	Hypothetical	Conserved hypothetical protein	hypothetical protein bsr6521
blr7345	+1.8	0.00	Hypothetical	No similarity	hypothetical protein blr7345
bll1862	+1.8	0.45	Hypothetical	No similarity	hypothetical protein bll1862
bsl4593	+1.8	0.00	Hypothetical	No similarity	hypothetical protein bsl4593
bsr3925	+1.8	0.00	Hypothetical	No similarity	hypothetical protein bsr3925
bsl4650	+1.7	0.00	Hypothetical	No similarity	hypothetical protein bsl4650
bsr5316	+1.7	0.00	Hypothetical	Conserved hypothetical protein	hypothetical protein bsr5316
bsl1006	+1.6	0.00	Hypothetical	No similarity	hypothetical protein bsl1006
bsr6520	+1.6	0.45	Hypothetical	Conserved hypothetical protein	hypothetical protein bsr6520
bsl1845	+1.6	0.45	Hypothetical	No similarity	hypothetical protein bsl1845
bsr4821	+1.6	0.00	Hypothetical	No similarity	hypothetical protein bsr4821
bll7982	+1.6	0.00	Hypothetical	Conserved hypothetical protein	hypothetical protein bll7982
blr2140	+1.6	0.00	Hypothetical	Conserved hypothetical protein	hypothetical protein blr2140
blr4638	+1.6	0.00	Hypothetical	Conserved hypothetical protein	hypothetical protein blr4638
bsr4956	+1.6	0.00	Hypothetical	No similarity	hypothetical protein bsr4956
bll7221	+1.6	0.00	Hypothetical	No similarity	hypothetical protein bll7221
bll7990	+1.6	0.45	Hypothetical	No similarity	hypothetical protein bll7990
blr2456	+1.6	0.00	Hypothetical	Conserved hypothetical protein	hypothetical protein blr2456
blr4646	+1.5	0.45	Hypothetical	Conserved hypothetical protein	hypothetical protein blr4646
bll2471	+1.5	0.00	Hypothetical	Conserved hypothetical protein	hypothetical protein bll2471

Table S1. *Cont.*

Locus ID	Fold Change	*q*-Value (%)	Functional Level I	Functional Level II	Functional Level III (Description)
blr6074	+1.5	0.00	Hypothetical	Conserved hypothetical protein	hypothetical protein blr6074
bsr5273	+1.5	0.00	Hypothetical	No similarity	hypothetical protein bsr5273
blr4240	+1.5	0.00	Hypothetical	Conserved hypothetical protein	hypothetical protein blr4240
blr1854	+1.5	0.45	Hypothetical	Conserved hypothetical protein	hypothetical protein blr1854
bsr7720	+1.5	0.45	Hypothetical	No similarity	hypothetical protein bsr7720
blr7860	−3.3	0.00	Hypothetical	Conserved hypothetical protein	hypothetical protein blr7860
blr4417	−1.8	0.45	Hypothetical	No similarity	hypothetical protein blr4417
bll1406	−1.6	0.00	Hypothetical	Conserved hypothetical protein	hypothetical protein bll1406
